# Effects of Six-Week *Ginkgo biloba* Supplementation on Aerobic Performance, Blood Pro/Antioxidant Balance, and Serum Brain-Derived Neurotrophic Factor in Physically Active Men

**DOI:** 10.3390/nu9080803

**Published:** 2017-07-26

**Authors:** Ewa Sadowska-Krępa, Barbara Kłapcińska, Ilona Pokora, Przemysław Domaszewski, Katarzyna Kempa, Tomasz Podgórski

**Affiliations:** 1 Department of Physiological and Medical Sciences, The Jerzy Kukuczka Academy of Physical Education, 40-065 Katowice, Poland; b.klapcinska@awf.katowice.pl (B.K.); i.pokora@awf.katowice.pl (I.P.); k.kempa@awf.katowice.pl (K.K.); 2 Department of Tourism and Health Promotion, University of Technology, Faculty of Physical Education and Physiotherapy, 45-758 Opole, Poland; domaszewskiprzemek@wp.pl; 3 Department of Biochemistry, Poznan University of Physical Education, 61-871 Poznań, Poland; podgorski@awf.poznan.pl

**Keywords:** dietary supplements, *Ginkgo biloba* extract, antioxidant capacity, BDNF

## Abstract

Extracts of *Ginkgo biloba* leaves, a natural source of flavonoids and polyphenolic compounds, are commonly used as therapeutic agents for the improvement of both cognitive and physiological performance. The present study was aimed to test the effects of a six-week supplementation with 160 mg/day of a standardized extract of *Ginkgo biloba* or a matching placebo on aerobic performance, blood antioxidant capacity, and brain-derived neurotrophic factor (BDNF) level in healthy, physically active young men, randomly allocated to two groups (*n* = 9 each). At baseline, as well as on the day following the treatment, the participants performed an incremental cycling test for the assessment of maximal oxygen uptake. Venous blood samples taken at rest, then immediately post-test and following 1 h of recovery, were analyzed for activities of antioxidant enzymes and plasma concentrations of non-enzymatic antioxidants, total phenolics, uric acid, lipid peroxidation products, ferric reducing ability of plasma (FRAP), and serum brain-derived neurotrophic factor (BDNF). Our results show that six weeks’ supplementation with *Ginkgo biloba* extract in physically active young men may provide some marginal improvements in their endurance performance expressed as VO_2_max and blood antioxidant capacity, as evidenced by specific biomarkers, and elicit somewhat better neuroprotection through increased exercise-induced production of BDNF.

## 1. Introduction

Several epidemiological studies have evidenced that high dietary intake of natural polyphenolic compounds synthesized by plants is associated with lower incidence of several chronic diseases associated with increased oxidative stress in humans [1,2]. It should be stressed that dietary flavonoids and other polyphenolic compounds can stimulate the expression of antioxidant enzyme genes and may limit, or even prevent, exercise-induced tissue damage. The most promising health benefits associated with the intake of foods rich in natural polyphenols include a reduced risk of coronary heart disease, and prevention of cancer and some neurodegenerative diseases [1,2,3]. The discrepancies in findings among several studies suggest that many polyphenols may have also pro-oxidant activities both in vitro and in vivo [4,5]. Although polyphenols are believed to be highly important for human health, they do not meet the classical criteria of essential nutrients because they are not necessary for growth and development. However, as they are essential for reaching the full lifespan, they have been termed “lifespan essential” [6]. Standardized *Ginkgo biloba* extract (EGb) includes over 60 bioactive components, the most important being flavonoids and terpene lactones [7,8]. Due to their potent antioxidant properties, flavonoids, such as quercetin and kaempferol, can directly quench free radicals; however, through induction of cytochrome P450 enzyme system activity, they can also indirectly reduce free radical formation [4,9,10]. However, flavonoid bioavailability is rather low due to their poor absorption. They are not absorbed intact by the intestinal mucosa as, prior to absorption, they undergo enzymatic conversion by the natural flora of the small intestine. The resultant aglycones are absorbed into the blood stream and, along with albumines, are transported to the liver. There, in the presence of phase I and phase II enzymes, they are metabolized to secondary metabolites transported to the tissues or eliminated in bile and urine [3,11]. Maximal concentrations of polyphenols in human plasma is usually reached about 1.5 to 5.5 h after consumption of polyphenol-rich foods [12]. Of note, maximum concentration of quercetin conjugates (the main flavonoids present in ginkgo [13]); in the plasma is reached 9 h after ingestion and its elimination is relatively slow (with a half-life of 24 h), possibly due to its high affinity for plasma albumin. Yet, the elimination half-life period for most flavonoids absorbed in the small intestine is much shorter (1–2 h) [11,14]. Terpene lactones (such as ginkgolides and bilobalide) possess very specific and potent antagonist activity against platelet aggregation factor (PAF). They facilitate blood flow through the cerebrum, dilate the capillary vessels, have protective effects on myelin sheaths, prevent thrombosis, and boost concentration and the learning process [15]. EGb supplementation has been shown to have a neuroprotective effect and capacity to improve cognitive functions by decreasing oxidative stress and increasing the concentration of brain-derived neurotrophic factor (BDNF) [16]. BDNF is a molecular mediator of synaptic plasticity, hence, the BDNF signaling pathway is reduced in many neurodegenerative and psychiatric diseases [17]. Of note, BDNF expression is augmented by physical exercise [18].

Taking into account a growing interest in the use of natural herbal supplements, the aim of this study was to examine the effect of *Ginkgo biloba* supplementation on aerobic performance, blood pro/antioxidant balance, and the level of BDNF in physically active men.

## 2. Materials and Methods

### 2.1. Participants

Eighteen healthy (as evidenced by medical certificate), non-smoking male physical education students (aged 20–24 years) volunteered to participate in this study. All individuals were informed about the purpose and nature of the experiment before giving their written consent to participate. The study protocol conformed to the ethical guidelines of the World Medical Association Declaration of Helsinki, was approved by the Institutional Ethics Committee at the Jerzy Kukuczka Academy of Physical Education in Katowice (certificate of approval No. 4/2013). The exclusion criteria were the use of tobacco products, alcohol consumption, and the use of any medicine or dietary supplements during the four weeks prior to the study.

The participants were randomly allocated to two groups: (1) the control placebo group (*n* = 9, PLA); and (2) the study group (*n* = 9, EGb) supplemented with standardized *Ginkgo biloba* extract, in the form of soft gelatinous capsules (Ginkoflav^®^ forte, Olimp Labs, Dębica, Poland) administered at a dose of two capsules once a day after breakfast for six weeks. EGb capsules contained 80 mg of standardized *Ginkgo biloba* extract containing 19.2 mg flavonoid glycosides (24%), 4.8 mg terpene lactones (6%), and additional substances, such as maltodextrin, microcrystalline cellulose, and magnesium stearate. Placebo capsules contained microcrystalline cellulose, magnesium stearate, and maltodextrin instead of Ginkgo plant extract. There were no dropouts from the study and all participants completed the full survey.

### 2.2. Study Design

All subjects performed an incremental test on a cycle ergometer (Sport Excalibur, Groningen, The Netherlands) while connected to a breath-by-breath gas analyzer (MetaLyzer 3B-R2, Leipzig, Germany) to determine maximal oxygen uptake (VO_2_max). After a short warm-up cycling at 20 W, the subjects cycled in stages of 3 min duration starting at a power output of 40 W, increasing progressively by 40 W, and continuing until exhaustion. The students participated in the exercise test twice, i.e., before the start (first trial) and after six weeks of supplementation with EGb or PLA (second trial). In each trial, venous blood samples from the antecubital vein were drawn into tubes without anticoagulant or into the heparinized test tubes at rest, then at 3 min post-test, and again after 1 h of recovery.

### 2.3. Biochemical Analyses

Samples of venous blood were collected before starting and on the day following the completion of supplementation with EGb or PLA. Fresh whole blood samples were immediately assayed for reduced glutathione (GSH) by a colorimetric method with 5,5′-dithiobis-2-nitrobenzoic acid [19], or collected in test tubes for the separation of serum or plasma. A portion of heparinized blood was centrifuged for 10 min at 1000× *g* at 4 °C to separate plasma and erythrocytes that were then washed three times with cold saline (4 °C) and kept frozen at −80 °C (for a period not longer than one month, without repeated freezing and thawing) until analysis for activities of red blood cell antioxidant enzymes, i.e., superoxide dismutase (SOD, EC 1.15.1.1) using the commercially available RANSOD SD125 kit (Randox Laboratories Ltd., Crumlin, UK); glutathione peroxidase (GSH-Px, EC 1.11.1.9) with the commercial RANSEL RS505 kit (Randox Laboratories Ltd., Crumlin, UK), catalase (CAT, EC 1.11.1.6) by the method of Aebi [20], and glutathione reductase (GR, EC 1.6.4.2) according to Glatzle et al. [21]. The activities of all antioxidant enzymes were measured at 37 °C and expressed per 1 g of haemoglobin as assayed by a standard cyanmethemoglobin method using a diagnostic kit No. HG980 (Randox Laboratories Ltd., Crumlin, UK). Fresh plasma samples were assayed for the concentration of uric acid (UA) using diagnostic kits from Randox Laboratories (UA230). The total antioxidant capacity of plasma was assessed using the ferric reducing ability of plasma (FRAP) assay according to Benzie and Strain [22]. Assessment of plasma lipid peroxidation was conducted using the thiobarbituric acid (TBARS) reaction according to Buege and Aust [23]. Plasma concentration of total phenolics was measured by the colorimetric method [24]. The serum level of brain-derived neurotrophic factor (BDNF) was determined by the immunoenzymatic method using a diagnostic RayBioHuman^®^ BDNF ELISA kit No. ELH-BDNF-001 (RayBiotech, Inc., Norcross, GA, USA). Biochemical analyses were performed in our certified laboratory fulfilling the requirements of PN EN-ISO 9001:2009 (certificate No. 129/2015) according to instructions provided by the manufacturers of laboratory tests used in this study.

### 2.4. Statistical Analysis

Data presented as means (±SD) were calculated for all variables. A three-way repeated-measures ANOVA with two groups (PLA and EGb) × two trials (at the start—“First trial”—and the end—“Second trial”—of the experiment) × three time-points (at rest, 3 min post-test, and post-1 h recovery) as the main factors followed, when appropriate, by the Bonferroni post hoc tests, which were used to test the significance of between group differences. All statistical analyses were computed using Statistica 10.0. software (StatSoft, Tulsa, OK, USA). The level of significance was set at *p* < 0.05.

## 3. Results

### 3.1. Subjects

All participants not involved in sports activities other than those scheduled at the faculty were characterized by comparable baseline maximal oxygen uptake (VO_2_max) within the range of 40 to 55 mL O_2_/min/kg_BW_, rated as average or above average for their age category (18–25 years), thus they may be categorized as active college-aged males. No significant intergroup differences were found in somatic parameters and peak VO_2_ values. The basic characteristics of participants are presented in Table 1.

### 3.2. Antioxidant Capacities

**Figure 1:**
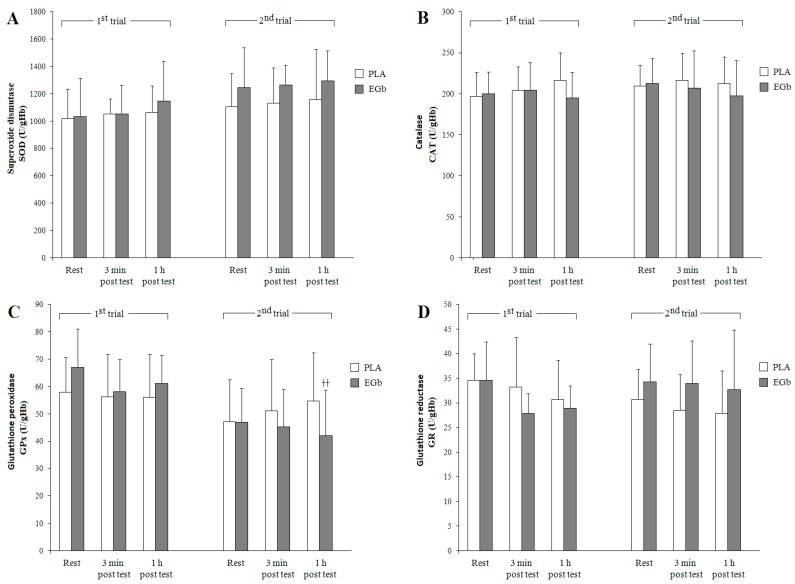
Changes in blood antioxidant enzymes activities in response to dietary supplementation and exercise. Note: Data are means ± SD; significant differences: ^††^ *p* < 0.01 vs. respective values before treatment. PLA: placebo, EGb: *Ginkgo biloba* extract. SOD: superoxide dismutase (**A**); CAT: catalase (**B**); GPx: glutathione peroxidase (**C**); and GR: glutathione reductase (**D**).

**Figure 2:**
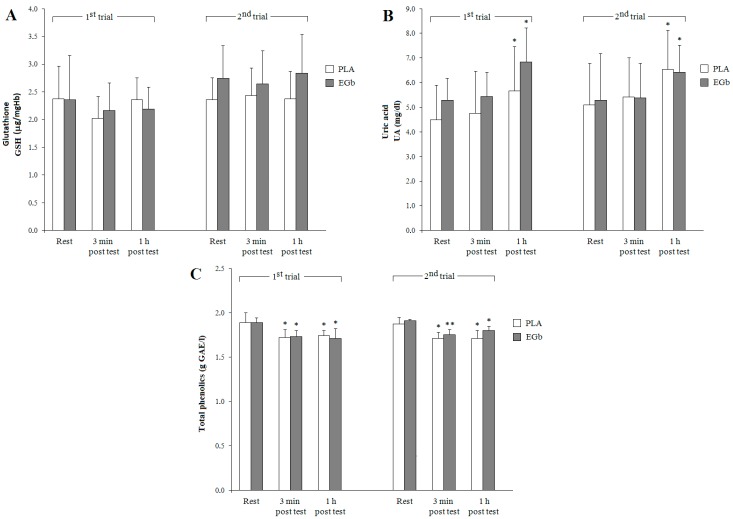
Changes in blood non-enzymatic antioxidants concentrations in response to dietary supplementation and exercise. Note: data are means ± SD; significant differences: * *p* < 0.05, ** *p* < 0.001 vs. resting values. PLA: placebo, EGb: *Ginkgo biloba* extract, GSH: reduced glutathione (**A**); UA: uric acid (**B**); and total phenolics (**C**).

The results of biochemical analyses of the studied enzymatic and non-enzymatic antioxidant defense components are presented, respectively, in Figure 1 and Figure 2. The activities of SOD and CAT were not significantly affected by supplementation with *Ginkgo biloba* extract, although SOD activities recorded in both groups at rest and post-exercise were slightly higher compared to the corresponding baseline values. A repeated measures three-way ANOVA revealed a significant trial effect (F = 4.49, *p* < 0.05) on SOD. In contrast, GPx activity in both groups was lower in the second compared to the first trial, as evidenced by a significant trial effect (F = 15.24, *p* < 0.001), although larger declines were recorded in the EGb group, also revealed by the trial × group interaction close to significance (F = 3.84, *p* = 0.07). There were also significant time-effect (F = 5.21, *p* < 0.01) and trial × group interaction (F = 5.09, *p* < 0.05) on GR activity.

Despite the lack of significant differences between the pre- and post-exercise GSH concentrations, significant trial (F = 11.38, *p* < 0.001) and trial × group interaction effects (F = 4.87, *p* < 0.05) as well as close to significance time-effect (F = 2.64, *p* = 0.09) on GSH were noted (see Figure 2). Supplementation with EGb did not affect the UA level, although in both the placebo (PLA) and EGb-supplemented groups, the exercise test induced marked increases in UA levels at 1 h of recovery compared to the respective resting values, as reflected by the highly significant time-effect (F = 36.77, *p* < 0.0001). Similarly, supplementation with *Ginkgo biloba* extract did not affect the total phenolics level, however, in both groups (PLA and EGb) their content decreased immediately after completion of the graded exercise test compared to resting values. The significant time-effect (F = 39.64, *p* < 0.0001) and close to significance trial × group interaction (F = 3.46, *p* = 0.08) were revealed by three-way ANOVA.

**Figure 3:**
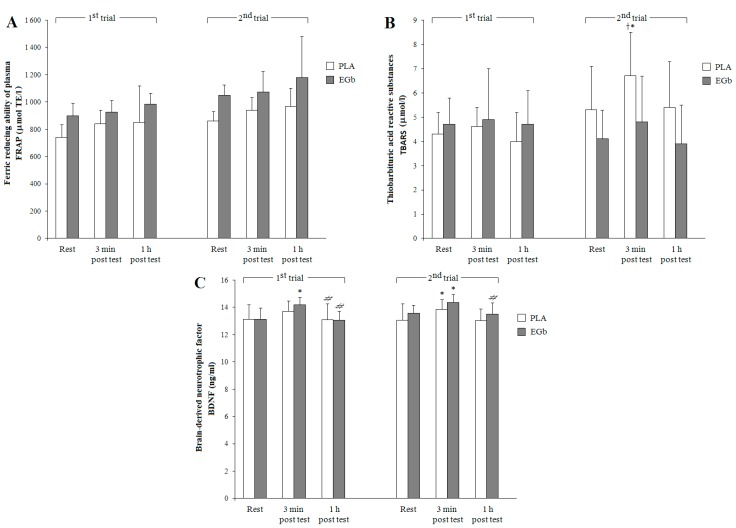
Plasma total blood antioxidant status (FRAP) and lipid peroxidation products (TBARS) level and serum brain-derived neurotrophic factor (BDNF) in response to dietary supplementation and graded exercise. Note: data are means ± SD; significant differences: * *p* < 0.05 vs. resting values; ^#^ *p* < 0.05 vs. values immediately post test; ^†^ *p* < 0.05 vs. respective values before treatment. PLA: placebo, EGb: *Ginkgo biloba* extract. FRAP: ferric reducing ability of plasma (**A**); TBARS: thiobarbituric acid reactive substances (**B**); BDNF: brain-derived neurotrophic factor (**C**).

Data regarding antioxidant status evaluated using FRAP assay and lipid peroxidation using the TBARS method, as well as serum BDNF content are presented in Figure 3.

Despite the lack of significant differences between the pre- and post-exercise FRAP levels, a three-way ANOVA revealed significant effects of group (F = 10.37, *p* < 0.01), trial (F = 13.64, *p* < 0.001), and time (F = 16.29, *p* < 0.0001). Increased levels of TBARS, compared to resting values, were recorded immediately after a graded exercise test in both groups, however, significant differences were found only in the PLA group. After a six-week long EGb supplementation, a moderate decrease in resting and post-exercise TBARS levels, compared to respective values recorded in the first trial, was observed. Significant effects of trial (F = 7.08, *p* < 0.05), time (F = 8.03, *p* < 0.001), and a very close to significance trial × time interaction (F = 3.09, *p* = 0.06) on TBARS levels were revealed by three-way ANOVA.

Before and following a six-week PLA or EGb supplementation, a moderate increase in post-exercise serum BDNF compared to resting values was observed, followed by its further decline to basal levels over the recovery. Three-way ANOVA showed significant effects of trial (F = 5.15, *p* < 0.05), time (F = 27.23, *p* < 0.0001), and trial × group interaction (F = 4.57, *p* < 0.05) on serum BDNF.

## 4. Discussion

The main focus of this study was to test whether a six-week supplementation with *Ginkgo biloba* extract would modify aerobic performance, improve blood antioxidant capacity, and enhance BDNF expression in healthy, physically active young men. Previous human studies reported a positive correlation between the increase in endurance capacity (VO_2_max) and the consumption of selected herbal supplements rich in polyphenols (i.e., quercetin or epigallocatechin-3-gallate) in healthy, untrained volunteers [25,26]. Flavonoids and terpenes contained in *Ginkgo biloba* extract can stimulate the release of endothelium-derived relaxing factor (EDRF), which may increase muscle tissue blood flow through improved microcirculation and can thus improve aerobic endurance by enhancing muscular energy production [27]. Of note, some improvement of exercise performance evaluated by pain-free walking distance was reported in *Ginkgo biloba*-treated patients with peripheral occlusive arterial disease (POAD) [28], although the efficacy of this treatment is still discussed [29]. Statistically significant, although trivial improvements in VO_2_max ranging from 2.3 to 7.5% were reported after five days of supplementation with quercetin (500 to 1000 mg/d) in untrained, active, or moderately-trained individuals with VO_2_max levels comparable to those reported in our study [25,30,31]. It seems that the dosage of herbal supplement may be a factor determining aerobic power. Mahady [32] showed that recommended dose of EGb standardized extract to achieve beneficial effects is located in a dose between 40 and 60 mg of EGb 3–4 times daily. In our study, subjects consumed 160 mg of EGb extract once a day for six weeks, which may explain only a marginal (6% vs*.* 1%) increase in the relative percentage VO_2_max change (baseline to intervention) scores in individuals receiving, respectively, the EGb or the placebo capsules.

The multifaceted activities of *Ginkgo biloba* extract may also derive from other mechanisms of action, such as their antioxidant potential. Antioxidant effects are connected with a capacity of scavenging reactive oxygen species (ROS) and a possibility of increasing activities of antioxidant enzymes, such as superoxide dismutase (SOD), glutathione peroxidase (GPx), catalase (CAT), and heme-oxygenase-1 by upregulating the expression of antioxidant genes encoding these enzymes [16,33].

First, we decided to evaluate the activities of the main antioxidant enzymes, i.e., SOD, CAT, GPx, and GR, in the blood. Although the statistical analysis did not reveal significant changes in antioxidant enzymes between the first and the second trials, a 20% rise in resting SOD activity was observed after EGb supplementation (reflected by significant trial effect), compared with an 8% increase found in the placebo group. Superoxide dismutase is the most important antioxidant enzyme, as it catalyzes the dismutation reaction resulting in the elimination of superoxide anion radicals, thus preventing the formation of other reactive oxygen species [34]. The product of SOD-catalyzed reaction, hydrogen peroxide, is enzymatically decomposed to water and oxygen by catalase (CAT) and glutathione peroxidase (GPx), the latter with higher affinity to H_2_O_2_ than CAT [35]. Since the actions of enzymatic antioxidant defense systems are coordinated, compensatory changes in enzymatic activity might be expected, i.e., a change in the activity of one enzyme may affect the activity of another one. This might particularly concern the competition between GPx and CAT. Our study showed a marginal increase in resting CAT activity and a decrease in GPx in both EGb-supplemented and PLA control groups.

Several non-enzymatic antioxidants were also assessed, including reduced glutathione (GSH), uric acid (UA), and total phenolics. Supplementation with *Ginkgo biloba* extract did not significantly affect concentrations of total phenolics and UA. Only in the case of GSH, which presented slightly higher levels in the second trial (reflected by significant trial effect) in the EGb group, support the previous finding of Moskaug et al. [36], that dietary polyphenols can modulate the expression of the γ-glutamylcysteine synthetase-enzyme involved in GSH synthesis. Of note, significantly higher UA, but lower total phenolics levels, were observed in both groups (EGb and PLA) during the recovery post-graded exercise test. With respect to uric acid, a final product of the degradation of purine nucleosides under conditions of energy stress, when the rate of ATP utilization in skeletal muscle exceeds that of its resynthesis [37], and one of the main antioxidants in human plasma [38], its high levels are characteristic for body fluids, tissues, and cells under severe oxidative stress. As found in our study, neither a six-week EGb supplementation nor a graded exercise test affected the plasma uric acid level recorded immediately post-completion of this test, but in both experimental groups, its significant increase was observed during the recovery period; the main time-effect appeared highly significant. These results are consistent with those reported by Dudzińska et al. [39] and Hellsten-Westing et al. [40]*,* who found that plasma uric acid reached its peak value within an hour after completing a maximal effort, i.e., under the ischemia/reperfusion condition.

It is worth mentioning that the health effects of polyphenols derived from the dietary sources depend on their bioavailability. Importantly, blood samples for biochemical analyses in our study were taken one day after ingesting the last EGb or PLA capsule, which may explain the lack of marked between-group differences in plasma total phenolics levels under resting conditions. We may also refer to the recent report on the dietary intakes estimated for the representative population of Polish adults, in which the calculated daily total polyphenol intake was 989 mg, with phenolic acids and flavonoids as the main contributors [41]. This may explain that the dose contained in *Ginkgo biloba* capsule had little impact on plasma level of total phenolics assessed one day after ingestion of the last EGb capsule. In the present study, independent of the subjects’ group and trial, plasma total phenolics were significantly decreased 3 min after completion of the graded exercise test, as reflected by a highly significant time-effect. These results may imply that the plasma polyphenols have been used for scavenging free radicals produced during the exercise test.

It should be stressed, however, that although the antioxidant activity of dietary polyphenols has been well documented in vitro, there is much more doubt about their antioxidant action in vivo*.* Therefore, considering that polyphenols derived from the diet are present in the circulation and tissues only in low micromolar ranges, it is far more likely that they may activate signaling pathways for the production of cytoprotective enzymes or metabolites [1,10,36,42]. Of note, a major mechanism of action for certain nutritional antioxidant phytochemicals found naturally in plants is the paradoxical oxidative activation of the NF-E2-related factor 2 (Nrf2) signaling pathway, which maintains protective antioxidant enzymes against oxidative damage and nucleophilic components of the antioxidant defense (such as GSH) in a reduced state [5,43]. This presumption is supported by our finding of some increases in SOD activity and GSH content (revealed by significant trial effects: F = 4.49, *p* < 0.05 and F = 11.38, *p* < 0.001, respectively) in EGb-supplemented individuals.

The adverse effects of exercise-induced oxidative stress were evaluated based on lipid peroxidation assessed by TBARS assay. As predicted, the post-exercise rise in lipid peroxidation levels, expressed as TBARS, was recorded in both groups, but apparently lower increases were found in the EGb-supplemented individuals. Our results appear to be similar to those reported by Jówko et al. [44] and Kuo et al. [9], who also found a decrease in post-exercise lipid peroxidation, expressed as malondialdehyde (MDA), following supplementation with natural polyphenol-rich extracts. It is noteworthy that dietary polyphenols may be incorporated into cell membranes, which results in a change in cell structure stability and better protection of cell membranes against lipid peroxidation [4]. The beneficial effect of EGb supplementation on antioxidant capacity was also supported by significant negative correlation between SOD and TBARS (R = −0.57, *p* < 0.01) in EGb-supplemented individuals. When interpreting these data, one should realize that the commonly used levels of TBARS or MDA as markers of lipid peroxidation are now considered somewhat controversial, although they are still usable with cautious interpretation [42].

It is well known that the coordination of antioxidant defense systems presents higher antioxidant potential than a separate antioxidant molecule or antioxidant enzyme. To solve this problem, we evaluated the plasma total antioxidant capacity assessed by the FRAP assay, which provides much more information on free radical scavenging capacity than the concentrations of individual antioxidants [45,46]. Our study revealed a moderate increase in resting and post-exercise FRAP levels after EGb supplementation, reflected by significant effects of group, trial, and time. The observed increases in FRAP levels during recovery may be attributed to a concurrent rise in plasma uric acid, which is the main contributor to total antioxidant capacity expressed as a FRAP value [22,45]. It should be emphasized that only in the EGb-supplemented group was FRAP highly significantly correlated with UA levels (R = 0.83, *p* < 0.0001).

Finally, we have to recall that polyphenols contained in *Ginkgo biloba* extract may provide other health benefits, such as neuroprotection regulated by neurotrophic factors, among which the best studied are nerve growth factor (NGF) and brain-derived neurotrophic factor (BDNF). The effect of regular physical activity on the structure and cognitive functions of the brain has been well documented by experimental and observational studies [47,48,49,50]. Regular physical exercise of moderate intensity has a favorable influence on brain vascularization and blood flow, thus improving oxygen and nutrient supply. Physical effort may enhance memory through neurogenesis, changes in the levels of neurotransmitters, and neurotrophic factors, primarily in the brain regions of the hippocampus, cerebral cortex, hypothalamus, and cerebellum [51,52]*.* Potential synergy between dietary polyphenol consumption and physical activity may also involve signaling pathways regulating different stages of neurogenesis, cell fate, synaptic plasticity, cognitive performance, and blood vessel function [53]. Hence, a combination of active lifestyle and dietary supplements of natural origin seems to be an optimal strategy for improving or maintaining brain function.

Our results have evidenced that a six-week consumption of *Ginkgo biloba* extract did not result in an increase in basal BDNF content. However, during the incremental test to exhaustion, significant increases in serum BDNF concentration immediately post-test and its rapid decline to basal values were observed in both groups (EGb and PLA), although higher post-test BDNF increases were recorded in EGb-supplemented individuals (revealed by significant group × trial interaction)***.*** In this context, our data are similar to those reported by Bell et al. [13] and Rojas Vega et al. [54] for male individuals performing a short-duration incremental cycling exercise to exhaustion.

The main limitations of this study were the relatively small number of participants recruited from a population of physically active college-aged males, and the lack of information on their actual food intakes and physical workloads, which limits the generalizability of the research findings to a wider population. Other weakness of this study is that we could not perform more specific and more reliable diagnostic tests for the determination of the plasma total antioxidant capacity and lipid peroxidation.

## 5. Conclusions

Our results show that six weeks’ supplementation with a standardized *Ginkgo biloba* extract in physically active young men may provide some marginal improvements in their endurance performance expressed as VO_2_max and blood antioxidant capacity, as evidenced by specific biomarkers (SOD, GSH, UA, FRAP, and TBARS). Moreover, it may provide somewhat better neuroprotection through increased exercise-induced production of BDNF.

## Figures and Tables

**Table 1:** Basic characteristics of the participants at pre- and post-treatment stages.

Group	Age (Years)	Height (cm)	Body Weight (kg)	VO_2_max (mL/min/kg)	
				First Trial	Second Trial
PLA	22.3 ± 1.1	179.7 ± 7.8	79.7 ± 10.8	45.7 ± 5.1	46.1 ± 4.1
EGb	22.5 ± 0.9	182.1 ± 5.9	80.1 ± 6.8	46.2 ± 4.8	48.9 ± 5.8

Note: Data are means ± SD, PLA: placebo, EGb: *Ginkgo biloba* extract.
